# Non-Alcoholic Fatty Liver Disease and Its Association With Diabetes Mellitus

**DOI:** 10.7759/cureus.17321

**Published:** 2021-08-20

**Authors:** Jaskamal Padda, Khizer Khalid, Anwar Khedr, Fahriba Tasnim, Ola A Al-Ewaidat, Ayden Charlene Cooper, Gutteridge Jean-Charles

**Affiliations:** 1 Internal Medicine, JC Medical Center, Orlando, USA; 2 Internal Medicine, Advent Health & Orlando Health Hospital, Orlando, USA

**Keywords:** non-alcoholic fatty liver disease, diabetes mellitus, insulin resistance, oral hypoglycemics, hepatic steatosis, hepatocellular carcinoma, nash, bariatric surgery

## Abstract

There is a bidirectional relationship between non-alcoholic fatty liver disease (NAFLD) and type 2 diabetes mellitus (T2DM). The liver has a vital role in the pathophysiology of both diseases as it leads to the development of insulin resistance (IR), which in turn results in NAFLD and T2DM. It has been shown that T2DM increases the risk of NAFLD progression. Furthermore, the presence of NAFLD raises the probability of T2DM complications, which explains the increased rates of NAFLD screening in patients with T2DM. In addition, there are common management options for the two diseases. Lifestyle changes can play a role in the initial management of both diseases. Medications that are used to treat T2DM are also used in the management of NAFLD, such as metformin, thiazolidinediones (TZD), glucagon-like peptide-1 (GLP-1) analogues, and dipeptidyl peptidase-4 (DPP4) inhibitors. Bariatric surgery is often used as a last resort and has shown promising results. Lifestyle interventions with diet and exercise are important postoperatively to maintain the weight loss. There are many novel treatments that are being investigated for the treatment of NAFLD, targeting multiple pathophysiologic pathways. This review aims to shed some light on the intricate relationship between NAFLD and T2DM and how IR links both diseases. We also try to raise awareness among clinicians about this relationship and how the presence of one disease should raise a high index of suspicion for the existence of the other.

## Introduction and background

Non-alcoholic fatty liver disease (NAFLD) is a range of liver disorders that includes hepatic steatosis, steatohepatitis, and hepatic fibrosis. This may further progress to cirrhosis and hepatocellular carcinoma (HCC) [1]. It is caused by fatty infiltration of more than 5% of liver hepatocytes, in the absence of a known cause of fatty liver disease, such as significant alcohol use, medications, or genetic disorders [2]. Approximately 25% of the global population has been affected by NAFLD [3]. Middle Eastern countries and South America account for the highest prevalence of NAFLD globally [4]. In the United States, it has been the major cause of chronic liver disease and around 90 million people are affected. Nearly one-quarter of them progresses to non-alcoholic steatohepatitis (NASH) [5]. The prevalence of NAFLD has been increasing progressively over the past years due to the higher prevalence of obesity, physical inactivity, metabolic syndromes, and type 2 diabetes mellitus (T2DM), which represent the major risk factors of NAFLD. It was found that more than half the patients with T2DM are diagnosed with NAFLD, which shows a strong relationship between them [6,7]. Furthermore, studies have shown that T2DM plays a major role in disease progression to NASH, fibrosis, and cirrhosis [8]. The majority of NAFLD patients are asymptomatic, and it is usually found incidentally during routine blood workups [9]. Although the gold standard diagnostic method for NAFLD is a liver biopsy, other, alternative, and non-invasive modalities have also been described, which include serum biomarkers and imaging studies. While ultrasound represents the main imaging study for NAFLD, CT and MRI have a diagnostic role as well [10]. However, reviewing the patient’s history to exclude alcohol use and other causes of liver disease represents a major step in the diagnostic criteria of NAFLD [11].

## Review

Pathophysiology of NAFLD

The Multi-Hit Theory

Although the pathogenesis of NAFLD has been investigated widely before, it is still not entirely understood. The most commonly proposed mechanism is the multi-hit theory. According to this, the first hit involves the build-up of triglycerides in the liver, causing steatosis. This increases the susceptibility of the liver to undergo second hits caused by oxidative stress, endoplasmic reticulum stress, mitochondrial dysfunction, inflammatory cytokines, adipokines, gut microbiota, and glucocorticoids [12,13]. The two-hit theory was modified to outline the role of free fatty acids (FFA) in causing liver injury through direct lipotoxicity. FFA influx into the liver leads to lysosomal destabilization, activating inflammatory pathways such as the nuclear factor kappa B-dependent tumor necrosis factor-alpha pathway [14]. The third hit is proposed to be initiated by the death of hepatocytes. The progression from non-alcoholic fatty liver to NASH occurs when the protective mechanisms of FFA-mediated lipotoxicity become exhausted, and the rate of hepatocyte death exceeds the rate of hepatocyte regeneration. This triggers the activation of myofibroblasts, which produce liver progenitor cells. These cells induce inflammatory immune responses and differentiate to replace the dead hepatocytes, causing variable degrees of hepatic architecture distortion [15].

The Role of Insulin Resistance

**Figure 1:**
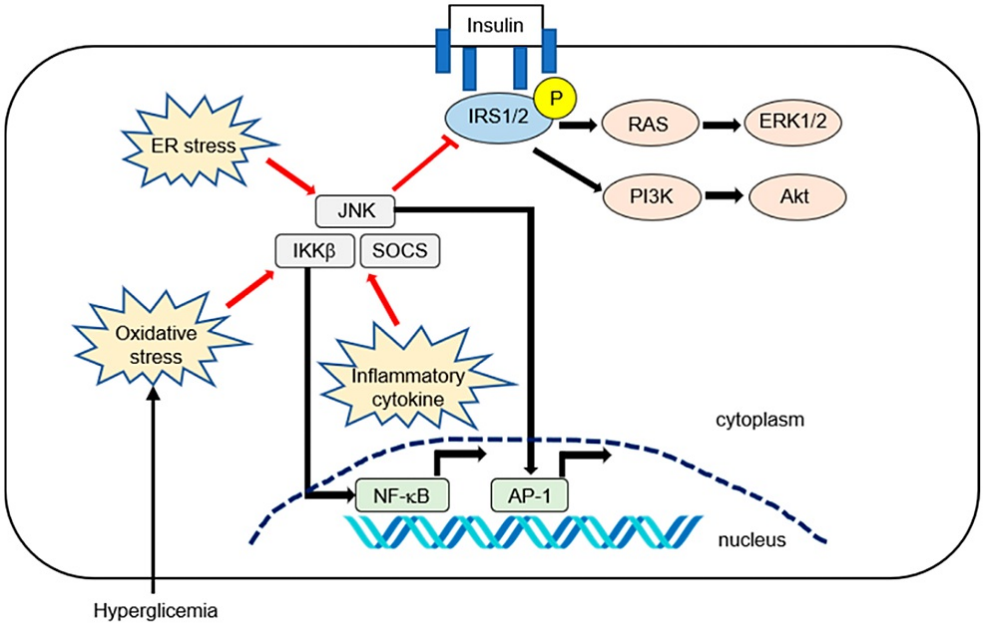
Summary of the inflammatory pathways affecting hepatic insulin resistance in NAFLD Insulin activates its receptor, which results in tyrosine phosphorylation of the IRS1 and IRS2 and activation of downstream effector pathways, including the PI3K-phosphoinositide-dependent kinase-AKT and the RAS-ERK pathways. Pro-inflammatory signaling or reactive oxygen species can activate IKK-β. The activated NF-kB is then translocated into the nucleus and binds to specific deoxyribonucleic acid response elements. Inflammatory cytokines such as interleukin-6 promote insulin resistance by inducing SOCS1 and SOCS3. SOCS1 and SOCS3 impair insulin signaling through ubiquitin-dependent degradation of IRS. The JNK, or mitogen-activated protein kinase, represents another important inhibitory kinase of IRS that is activated in response to a variety of extracellular stimuli and cellular stressors such as oxidative and ER stress ER: endoplasmic reticulum; JNK: c-Jun N-terminal kinase; IKK-β: IkappaB kinase beta; SOCS: suppressor of cytokine signaling; NF-κB: nuclear factor kappa-light-chain-enhancer of activated B cells; AP-1: activator protein 1; IRS1: insulin receptor substrate 1; IRS2: insulin receptor substrate 2; PI3K: phosphatidylinositol 3-kinase; AKT: protein kinase B; RAS: rat sarcoma; ERK1/2: extracellular-signal-regulated kinase 1/2 Copyright/license: this figure is from an open-access article distributed under the terms and conditions of the Creative Commons Attribution (CC BY) license (http://creativecommons.org/licenses/by/4.0/). No modifications were made to the original figure Fujii H, Kawada N, Japan Study Group Of Nafld J-N: The role of insulin resistance and diabetes in nonalcoholic fatty liver disease. Int J Mol Sci. 2020, 21:3863. 10.3390/ijms21113863 [18]

The most important link between NAFLD and T2DM is insulin resistance (IR). IR is defined as a suboptimal response to insulin functions in different tissues [16]. Insulin-enhanced FFA transport to the liver, decreased B-oxidation of FFA, increased de novo lipogenesis due to activation of lipogenic enzymes via sterol receptor-binding protein 1c, and increased triglyceride synthesis through the Kennedy pathway arise from insulin's compromised capacity to regulate lipolysis in the liver, adipose tissue, and skeletal muscles in IR states [17]. In these states, the beta cells of the pancreas try to overcome it by secreting more insulin. Furthermore, insulin clearance is also suppressed in patients with T2DM. All of this results in hyperinsulinemia, which has been shown to cause hepatocellular ballooning and lobular inflammation [16]. IR is affected by many inflammatory pathways in NAFLD, which are summarized in Figure 1 [18].

NAFLD and Genetic Predisposition

Although there is a definite association between NAFLD and IR, it has been shown that there are some genetic variants that cause hepatic steatosis in the absence of IR. Primarily, the missense mutation isoleucine-to-methionine substitution at residue 148 within patatin-like phospholipase domain-containing protein 3 (PNPLA3) increases NAFLD progression and results in decreased hepatic triglyceride hydrolysis [19]. Another PNPLA3 mutation [PNPLA3 glutamic acid-to-lysine substitution at residue 434, single nucleotide polymorphisms (SNP) rs2294918] has an effect on the PNPLA3 phenotype by lowering hepatic messenger RNA and protein levels. The glutamic acid-to-lysine substitution at residue 167 variant (at SNP rs58542926) of the transmembrane 6 superfamily member 2 protein influences liver fat content. This variant has been linked to a 34% increase in liver fat level in its carriers. Lastly, a mutation in the gene encoding 1-acylglycerol-3-phosphate O-acyltransferase causes its shortage, which results in severe steatosis [20].

NAFLD increases the risk of diabetes mellitus (DM) incidence

Studies have shown that NAFLD is a well-defined risk factor for T2DM. Various Korean observational studies have shown this correlation by tracking the risk of T2DM in non-DM patients over a five-year period based on their NAFLD status [21]. As NAFLD is a reversible condition in its early stages, it was found that the risk of T2DM decreased in patients with resolved NAFLD status [22]. In addition, studies have shown that the sustainability of NAFLD status is a key factor in increasing the risk of T2DM. Therefore, only patients with persistent NAFLD over a considerable period of time have shown a higher risk of T2DM development [23]. To date, three previously published meta-analysis studies have shown the increased risk of T2DM in patients with NAFLD diagnosed by different diagnostic modalities including liver enzymes and imaging studies [24-26]. The most recent one was published in 2018, and in it, most NAFLD patients were diagnosed by imaging studies. It also showed that the severity of NAFLD is directly correlated to T2DM risk, which carries a future impact on the diagnosis and management of T2DM [26]. This study has been the most extensive and valuable in the literature, as imaging studies are considered to be more accurate and specific NAFLD diagnostic modalities than serum markers [27]. Although liver biopsy is still the gold standard diagnostic modality, there has been no meta-analysis of biopsy-diagnosed NAFLD due to the unavailability of such studies. Therefore, more efforts are needed to investigate this to prove the true causality between NAFLD and T2DM and to demonstrate the extent of T2DM risk caused by variable NAFLD stages. Furthermore, more studies are needed to be conducted on populations with diverse ethnicities, as the previous studies were done mostly on Asian populations [26]. Regardless of these limitations, the most recent recommendation is to screen patients with NAFLD for T2DM [27].

DM increases the risk of NAFLD incidence

T2DM is considered a well-known risk factor for NAFLD. Studies have shown a higher prevalence of NAFLD in DM patients [28]. A recent meta-analysis published in 2017 showed that the pooled prevalence of NAFLD in DM patients is around 60%. This may vary according to gender, BMI, and the presence of other chronic disorders [29]. In addition, patients who presented with NAFLD and T2DM have shown a greater risk of chronic liver disease, fibrosis, and cirrhosis compared to non-DM patients [30] Moreover, NAFLD in DM patients increases the risk of cardiovascular disease and other diabetic complications [31]. Due to the progressively increasing incidence of T2DM over the past years [32], more studies have been conducted to assess the significance of early screening and treatment of NAFLD in DM patients [33]. Also, it has been suggested that there is a potential advantage in screening DM patients for NAFLD using MRI and magnetic resonance elastography (MRE) [34]. However, other studies support the importance of the healthcare providers to be watchful for NAFLD and chronic liver disease development in DM patients with no need for regular screening [35].

NAFLD increases the risk of DM complications

Several studies indicate that the effect of NAFLD is not confined to liver-related complications anymore. Moreover, it has a major influence on worsening DM complications. A study comprising 102 patients with NAFLD demonstrated that the incidence of impaired fasting glucose, dyslipidemia with elevated triglycerides and/or hypercholesterolemia, and hypertension increased after they had been diagnosed with NAFLD, necessitating regular screening for metabolic disorders in NAFLD patients [36]. Another study consisting of 358 patients with NAFLD and 788 matched controls with a follow-up period of six years showed evidence of increased incidence of obesity, hypertension, hypertriglyceridemia, hypercholesterolemia, DM, and multiple metabolic disorders in the patients' group compared to the healthy controls [37]. Moreover, several studies have indicated that NAFLD is closely linked to an increased risk of chronic vascular complications of DM [38]. In 2017, Guo et al. identified a heightened prevalence of carotid and lower limb atherosclerotic plaques to be associated with ultrasound-diagnosed NAFLD in people with T2DM [39]. Moreover, another study comprising individuals with T2DM established that the prevalence of vascular diseases is higher in individuals with NAFLD compared with healthy people [40]. A meta-analysis also identified the association between NAFLD and incident cardiovascular disease events [41]. When it comes to renal complications of DM, several studies have consistently revealed that NAFLD is linked to chronic kidney disease (CKD) [42]. A study including patients with T2DM revealed that the risk of CKD or advanced diabetic neuropathy increases twofold in patients with NAFLD independent of other factors [43]. In another study consisting of patients with T2DM, an association was found to be present between NAFLD and increased risk of incident CKD independent of other factors (adjusted hazard ratio: 1.49, 95% CI: 1.1-2.2) [44]. Moreover, a cohort study revealed NAFLD to be associated with a nearly threefold increased risk of CKD even after adjusting for confounding variables [45]. On the other hand, several experiments indicate NAFLD to be linked to a heightened prevalence of distal symmetric polyneuropathy in individuals with type 1 diabetes mellitus (T1DM) or T2DM [46,47]. Although there have been multiple studies showing evidence of the worse impact of NAFLD in DM complications, further studies are needed to establish this relationship with more precision.

DM increases the risk of NAFLD progression

T2DM plays an important role in the progression of NAFLD as T2DM has been proven to augment the risk of NASH by approximately two to threefold [30]. Based on liver histology, studies reveal that about 80% of patients with T2DM exhibit NASH, and 30-40% of them exhibit advanced fibrosis [48]. It was shown in a study that NAFLD and T2DM were established to be present in patients with HCC [49]. Furthermore, the risk of developing HCC increases by two to threefold with the presence of DM alone [50]. Therefore, the advancement of NAFLD is driven by the presence of DM. Numerous patients who have NASH developing into HCC have been found to have T2DM and other metabolic disorders [51,52]. Some researchers consider NAFLD to be an overlooked complication of DM due to the close association betweenT2DM and the progression of NAFLD to NASH [53].

DM augments NAFLD in many ways. Fatty-acid release from adipose tissue is increased by IR [54]. The IR status often plays a significant role in the upregulation of the hepatic uptake of fatty acids [55]. Liver inflammation and fibrosis are induced by direct lipotoxicity caused by excessive hepatic FFA influx in DM patients [56,57]. Further damage is caused to the liver by the oxidative stress induced by oxidation and metabolism of excessive FFAs in the liver [58], which consequently elicits hepatocellular damage and apoptosis. Hepatocyte apoptosis and necrosis are then activated by hepatocellular injury [59]. Consequently, this causes stimulation of hepatic stellate cells, and ultimately hepatic fibrosis [60]. There has been further evidence that liver fibrosis can be promoted by IR because of the stimulation of lysyl oxidase-like 2, independent of hepatic stellate cell activity [61].

Diagnosis and screening

Diagnosis of NAFLD

The diagnosis of NAFLD is made when there is evidence of hepatic steatosis on imaging or biopsy, with the exclusion of significant alcohol consumption, and no other etiologies for hepatic steatosis or chronic liver diseases. NAFLD is usually diagnosed incidentally during testing or screening for other diseases, on finding abnormal liver enzymes or abnormal abdominal imaging outcomes [62,63]. However, a liver biopsy is required to reach a definitive diagnosis of NAFLD and also for grading and staging of the disease [64]. There are many investigative modalities that have been studied to either diagnose NAFLD or monitor its severity, including blood tests, imaging studies, liver biopsy, and clinical scoring systems.

Blood tests such as liver enzymes are not accurate biomarkers of NAFLD. Even though they can be the only abnormal findings in NAFLD patients, up to 80% of NAFLD patients can have normal liver enzymes [65]. Furthermore, it was found that histological findings are similar in patients with NAFLD with normal alanine aminotransferase (ALT) levels when compared to those with high ALT levels [66]. It was also found that 76% of patients with DM and normal ALT levels have a high prevalence of NAFLD [30]. Novel diagnostic markers are being studied to diagnose NAFLD, such as microRNA 22, with promising results so far [67]. Several other biomarkers have been studied to help with fibrosis staging in NAFLD. For example, it was found that elevated immunoglobulin A levels are found in NAFLD patients and can predict the progression to an advanced fibrosis stage [68]. In addition, clinical scoring systems such as the NAFLD fibrosis score can be used to identify patients with NAFLD who are at a high risk of advanced disease and require further assessment [69].

Ultrasound is recommended as the first-line imaging tool for diagnosing NAFLD [64] because of its ability to assess hepatic fat content (Table 1) [70,71]. Due to its safety profile, low cost, accessibility, and reliability, it is recommended as the imaging technique of choice for screening for fatty liver [72]. Another imaging technique is the controlled attenuation parameter (CAP) (Table 2) [73]. It is an ultrasound-based elastography technique used to detect the severity of liver steatosis. CAP has been found to have good sensitivity and specificity, but its diagnostic accuracy is questionable, according to a meta-analysis by Shi et al. [74]. CT can also be used as an initial technique to diagnose NAFLD [63]. However, there are certain limitations to CT, such as its high cost, radiation exposure, non-availability for routine use, and low sensitivity in detecting mildly elevated hepatic fat content [70]. Other imaging options are MRI-proton density fat fraction (MRI-PDFF) and magnetic reasoning spectrometry (MRS). MRI-PDFF has been found to correlate with the steatosis stage accurately, but it is suboptimal in detecting advanced fibrosis stages [75]. MRS is not recommended for use in the clinical setting [64], and its use is limited to research due to high cost, reduced accessibility, and the need for additional validation [76]. There are other imaging techniques that are used to diagnose fibrosis in NAFLD, such as transient elastography (FibroScan, Echosens™, Paris, France), MRE, and acoustic radiation force impulse imaging [63].

**Table 1:** Steatosis grading via ultrasound Ultrasound grading with descriptions of liver parenchyma and vessel appearance [71]

Score	Steatosis	Description of fatty infiltration in the liver	Visualization
0	Absent	Normal echotexture of the liver	Normal visualization of the portal vein wall
1	Mild	Slight/diffuse increase in liver echogenicity	Normal visualization of the portal vein wall
2	Moderate	Increase in liver echogenicity	Slightly impaired appearance of the portal vein wall
3	Severe	Increase in liver echogenicity	Poor/no visualization of the portal vein wall and posterior part of the right liver lobe

**Table 2:** CAP score CAP: controlled attenuation parameter; dB/m: decibels per meter [73]

Grade	CAP score	Fatty changes in the liver
S0: no steatosis	0–237 dB/m	0–10%
S1: mild steatosis	238–259 dB/m	11–33%
S2: moderate steatosis	260–292 dB/m	34–66%
S3: severe steatosis	>293 dB/m	>67%

There has been much debate about the routine use of liver biopsy for diagnosing NAFLD, considering its invasiveness, even though it remains the gold standard of diagnosis. It is recommended for confirming the diagnosis of NASH, monitoring patients with a high risk of disease progression, persistent elevation of liver enzymes for more than six months, excluding other causes of chronic liver diseases, and inconclusive imaging [63,64]. To diagnose NASH, three main histologic features are required: steatosis, lobular inflammation, and hepatocellular ballooning [64].

Should We Screen for NAFLD in Patients with T2DM?

Because of the high prevalence of NAFLD in patients with T2DM, the presence of T2DM should raise high suspicion for the simultaneous existence of NAFLD [77]. However, the American Gastroenterological Association does not recommend routine screening of NAFLD in patients with T2DM due to concerns pertaining to long-term benefits and cost-effectiveness [62]. There have been many studies that showed the benefits of screening for NAFLD in patients with T2DM. For example, in a prospective cross-sectional study, 679 patients with a confirmed diagnosis of T2DM were screened for NAFLD using transient elastography, and the findings showed a prevalence of 83.6% of NAFLD. In light of this, the authors suggested that there is an urgent need for NAFLD screening in patients with T2DM [78].

The role of lifestyle changes in the management of NAFLD and T2DM

Studies have shown a reciprocal relationship between T2DM and NAFLD, which means that they both can lead to each other [79]. In addition, T2DM and NAFLD share the same risk factors, which are mostly related to lifestyle habits. As a result, lifestyle changes have been considered as the first-line step in the prevention and treatment of NAFLD and T2DM. These lifestyle changes include diet, exercise, and weight loss [80]. With the progressively increasing incidence of NAFLD and T2DM over the past years, they have been considered a major risk factor for chronic liver disease and cardiovascular diseases. Thus, more efforts have been made to determine the exact effect of lifestyle changes on disease progression [81]. Diet changes include a low caloric diet in terms of carbohydrate and fat restriction [82]. Studies have shown that weight loss caused by a low carbohydrate diet has a more significant effect on NAFLD regression than a low caloric diet [83]. In addition, it has been shown that polyunsaturated fatty acids have a protecting effect on NAFLD [82]. However, high protein intake increases the risk of NAFLD [84]. Weight loss has a significant role in NAFLD and T2DM treatment. More than 5% weight loss is needed to reverse liver steatosis and around 10% is required for NASH regression [85]. Regarding physical activity, studies have shown that increased physical activity is associated with a remarkable improvement in liver function and other metabolic markers of the body, regardless of the patient's weight-loss status [86].

The role of oral hypoglycemics in the management of DM and how they affect the management of NAFLD

The main pathogenic mechanism of NAFLD is thought to be impaired response to insulin actions, or IR that leads to high amounts of FFAs and glucose in the blood, which consequently leads to accumulation of liver fat. For this reason, several experiments have been undertaken on the use of anti-diabetic drugs in patients with NAFLD [87].

Metformin

Four meta-analyses [88-91] were performed on various studies on the use of metformin in NAFLD. Although it demonstrated no major progress in liver histology, in the case of liver fibrosis, it revealed substantial improvement in blood cholesterol level, fasting plasma glucose, and hemoglobin A1c (HbA1c). These findings suggest that metformin can be useful as a treatment against NAFLD risk factors. Although the impact of NAFLD and IR on cancer development is widely recognized, metformin use appears to have a protective function against both hepatic [92,93] and non-hepatic cancers [94].

Thiazolidinediones (TZD)

Several meta-analyses [95-98] have been conducted to determine the role of TZD on liver histology in NASH patients, and the results have varied. All of them showed beneficial effects of TZD on lobular inflammation without any substantial improvement in the fibrosis of the liver. However, adding lifestyle modification to TZD yielded considerable improvement.

Glucagon-like Peptide-1 (GLP-1) Analogues

In an experiment where animals were treated with exenatide, a reduction in hepatic fat was identified [99-101]. However, whether human hepatocytes have GLP-1 receptors or how GLP-1 agonists act on the liver is still not clear [99-102]. According to the liraglutide safety and efficacy in patients with non-alcoholic steatohepatitis (LEAN) trial [103], liraglutide appreciably improved steatosis and hepatocyte ballooning, but there were no significant differences in either lobular inflammation or NAFLD Activity Score.

Sodium-Glucose Cotransporter 2 Inhibitors

Several pre-clinical studies performed with animal experimental models have demonstrated that canagliflozin, empagliflozin, luseogliflozin, tofogliflozin, ipragliflozin, and remogliflozin [104-112] could be linked to hepatic steatosis improvement. Moreover, another finding has stated that tofogliflozin possibly decreases the risk of advancement to HCC.

Dipeptidyl Peptidase-4 (DPP4) Inhibitors

A report in 2012 was the first to show an improvement in glycosylated hemoglobin as well as in hepatic steatosis on MRI in a 67-year-old Asian woman diagnosed with NAFLD who was treated with sitagliptin [113]. Furthermore, a randomized-controlled trial comprising 58 patients, who were randomly assigned to receive either placebo or vildagliptin 50 mg twice a day for 12 weeks, demonstrated significant improvement in BMI, cholesterol, aminotransferase, and triglyceride levels among those who took vildagliptin [114].

Sulfonylureas and Meglitinides

Sulfonylureas are typically contraindicated in chronic liver or renal disease patients due to their hepatic metabolism and renal excretion [115]. Meglitinides have a shorter half-life than sulfonylureas and they do not have considerable renal excretion. But it is still not clear whether repaglinide is safe to be used in DM patients with chronic liver disease [116,117].

The role of bariatric surgery in the management of NAFLD and T2DM

Bariatric surgery is often considered the last option in the treatment of obesity-related health conditions. Lifestyle interventions with diet and exercise are important postoperatively to maintain the weight loss [118]. Questionnaires can be used to assess adherence to diet and exercise. A questionnaire developed by Dubai et al. consisting of 14 questions proved to be a reliable tool to gauge adherence to lifestyle modification in 100 obese patients with NAFLD [119]. A number of studies have shown the positive impact of bariatric surgery in DM and NAFLD. The combination of Roux-en-Y gastric bypass (RYGB) and optimal medicine has been shown to improve HbA1c postoperatively [120,121]. After two years, patients who underwent RYGB had lower levels of fasting glucose compared to patients who received only medical therapy [120]. Patients undergoing vertical sleeve gastrectomy also had similar findings [121]. Moreover, once patients lose weight after bariatric surgery, IR gets improved, and it seems to correlate with improved C-reactive protein and tumor necrosis factor-α [122]. Additionally, there is also improvement in kidney damage as demonstrated by improved microalbuminuria [123].

There have also been significantly favorable outcomes when bariatric surgery was considered for proper candidates with NAFLD who have been unable to achieve adequate weight reduction by diet and lifestyle changes only. In a meta-analysis comprising 15 studies, 91% of patients had improvement in steatosis and 65.5% had decreased fibrosis [124]. Approximately 70% of patients had a total resolution of NASH. Most of these studies were prospective and utilized RYGB as the method of bariatric surgery. On the other hand, a study comprising seven morbidly obese patients with NAFLD demonstrated improvement in transforming growth factor-β1, α-smooth muscle actin, and inflammatory markers such as interleukin-8, which are considered as hepatic factors regulating fibrogenesis [125]. Several studies consisting of morbidly obese NAFLD patients have revealed positive outcomes with bariatric surgery: reduction in steatosis, lobular inflammation, ballooning, and fibrosis [126,127]. However, when it comes to the comparison between the efficacy of different types of bariatric surgery, RYGB appears to be superior to all others due to its excellent weight-loss effects [128]. In another one-year cohort study including morbidly obese NASH patients, 85% of patients had NASH resolution overall, but greater rates were accomplished after RYGB compared to adjustable gastric band (AGB) surgery [129]. Moreover, patients undergoing AGB had greater rates of persisting NASH compared to those who had RYGB [129]. 

Emerging treatments for NAFLD

Due to the many complex pathways involved in the pathogenesis of NAFLD, several therapeutic options have been investigated. In Table 3, we summarize some of the most significant interventional clinical trials with published results, which have tested therapies for NAFLD in adult patients [130-132].

**Table 3:** Summary of the most important clinical trials for new pharmacologic treatments for NAFLD ASK1: apoptosis signal-regulating kinase 1; FXR: farnesoid X receptor; NAFLD: non-alcoholic fatty liver disease; NASH: non-alcoholic steatohepatitis; F3, STELLAR III: patients with NASH and bridging fibrosis; F4, STELLAR IV: patients with compensated cirrhosis

Agent	Mechanism of action	Sponsor/status	Trial name/number/phase	Intervention	Population	Outcomes
Emricasan [130]	Pan-caspase inhibitor	Conatus Pharmaceuticals/completed	ENCORE-PH/NCT02960204/II	5 mg or 25 mg or 50 mg twice daily for 24 weeks compared to placebo	NASH-related cirrhosis and baseline hepatic venous pressure gradient ≥12 mmHg	No improvement in hepatic venous pressure gradient or clinical outcomes in patients with NASH-related cirrhosis and severe portal hypertension
Selonsortib [131]	ASK1 inhibitor	Gilead Sciences/terminated	Stellar III & IV/NCT03053050 & NCT03053063/III	6 mg or 18 mg daily for 48 weeks compared to placebo	F3, STELLAR III or F4, STELLAR IV	No antifibrotic effect in patients with bridging fibrosis or compensated cirrhosis due to NASH
Obeticholic acid [132]	FXR agonist	Intercept Pharmaceuticals/active	Regenerate/NCT02548351/III	10 mg or 25 mg daily for 18 months compared to placebo	Adult patients with definite NASH, NAFLD activity score of at least 4, and fibrosis stages F2-F3, or F1 with at least one accompanying comorbidity	Obeticholic acid 25 mg significantly improved fibrosis and key components of NASH disease activity among patients with NASH

## Conclusions

NAFLD and T2DM are considered as two different manifestations of the same pathology that is IR. The majority of chronic liver disease cases are caused by NAFLD. Due to its strong association with T2DM, it has been a hot research topic to further explore this relationship and its future clinical implications. NAFLD and T2DM are both considered risk factors for each other. This makes us attentive to the potential impact of early screening of one disease in the previous presence of the other. In addition, the coexistence of NAFLD and T2DM has synergistic effects on each disease’s progression and complications. NAFLD and T2DM have the same pathophysiology and almost the same risk factors. Therefore, they share the same prevention and management strategies, which mainly consist of lifestyle changes, the use of oral hypoglycemics, and bariatric surgery as the last-resort treatment modality, which has shown its effective impact on NAFLD and T2DM management. However, there have also been efforts to find novel treatment modalities for NAFLD, such as FXR agonists, ASK1 inhibitors, and pan-caspase inhibitors.
